# Screening for toxicity of azole fungicides in *Caenorhabditis elegans* identifies triflumizole as a potent reproductive and developmental toxicant

**DOI:** 10.17912/micropub.biology.001755

**Published:** 2025-08-07

**Authors:** Samantha Hughes, Charlotte Koopmans, Majorie van Duursen

**Affiliations:** 1 Amsterdam Institute for Life and Environment, Environmental Health and Toxicology, Vrije Universiteit Amsterdam, the Netherlands

## Abstract

Azoles are broad spectrum anti-fungal compounds that can disrupt steroid hormone synthesis, raising significant concerns regarding their impact on human development and reproduction. Chemical exposure in *Caenorhabditis elegans* may provide insights into the broader impact of azole fungicides relevant to human health. Exposure to triflumizole, resulted in a significant reduction in brood size as well as a dose-dependent decrease in worm development. Similarly, clotrimazole negatively impacted development, but not reproduction, while ketoconazole had no detrimental effect. Together this suggests further research is urgently needed to explore the impact of azole chemicals on human reproductive health.

**Figure 1. The impact of azole fungicides on reproduction and development in C. elegans .**
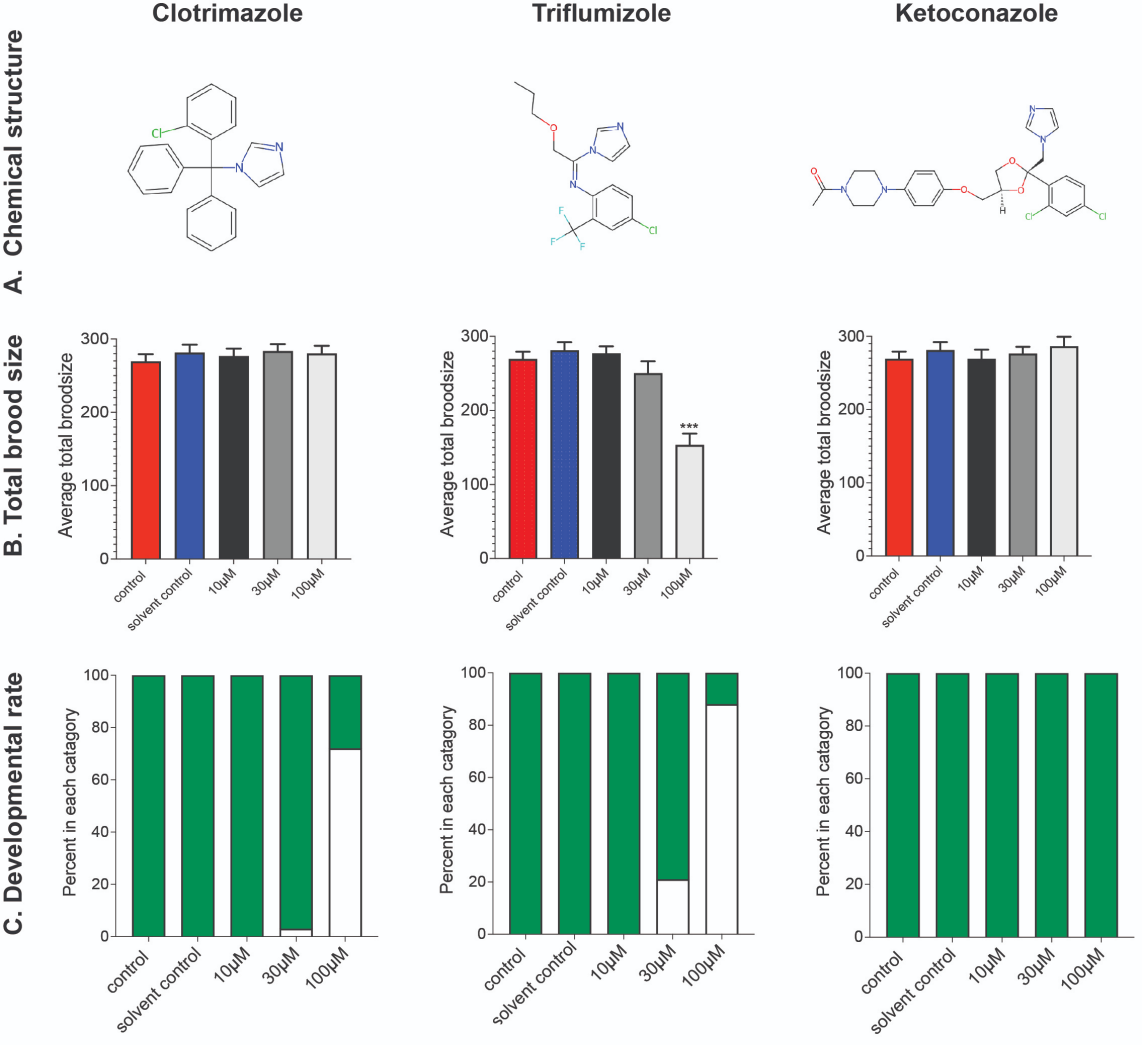
(A) Chemical structures of the 3 azoles tested. Structures downloaded from ChemSpider.com (B) Total brood size. The negative control is shown in red, and the vehicle control (0.2% methanol) is shown by the blue bars. Bars indicate averages with standard error of the mean. ***Statistically significantly difference from solvent control with *p* <0.001 (2-tailed t-test). *n* =19-24 over 2 independent biological replicates. (C) Developmental rate. Green bars indicate the percentage of surviving worms that have reached the L4 stage; white bars indicate the percentage of worms that have not reached L4 stage ( *n* =50-75).

## Description

Azole fungicides are broad spectrum anti-fungal compounds that are widely used as pesticides (Jørgensen & Heick, 2021), in pharmaceuticals and personal care products (Chen & Ying, 2015). The mode of action for azole fungicides is to inhibit fungal sterol 14α-demethylase CYP51, however, some azoles have the potential to inhibit mammalian enzymes including CYP19A/aromatase (Trösken et al., 2006; Trösken et al., 2004; Zarn et al., 2003). As a result, steroid hormone synthesis in mammals is disrupted, resulting in perturbation of sex steroid hormone homeostasis throughout early and late development, consequently providing a hazard for sexual development (Draskau & Svingen, 2022; Munkboel et al., 2019). In addition, azoles are able to target nuclear receptors, such as the estrogen and androgen receptors (Toporova & Balaguer, 2020), acting as potent antagonists to disrupt downstream processes (Draskau et al., 2021; Draskau et al., 2022; Jung et al., 2023). Together, this disruption can have consequences for fertility and reproductive function. Still, there are uncertainties regarding the developmental or reproductive toxicity (DART) of some azole fungicides, and more research is needed to fully explore the toxic potential of azoles.

Testing for DART endpoints typically requires many animals (e.g., rodents), which is costly, labour intensive and ethically challenging, thus an alternative approach is needed. *Caenorhabditis elegans* is a promising model for DART testing, aligning with the 3R principle and ethical considerations, and has previously been used for toxicological studies (Boyd et al., 2016; Boyd et al., 2012; Harlow et al., 2016; Leung et al., 2008). The use of whole-animal, non-vertebrate models, such as *C. elegans* aligns well with the European roadmap towards non-animal testing (Cronin et al., 2025) and the Adverse Outcome Pathway (AOP) framework, to explore the adverse effect in an intact organism or its progeny (Svingen, 2022).

The purpose of this study was to assess 3 azole fungicides (triflumizole, ketoconazole and clotrimazole; Figure 1A ) for their impact on development and reproduction using *C. elegans* . The agricultural fungicide triflumizole was chosen as, due to the highly lipophilic and persistent nature, it is frequently detected in the environment (Chen & Ying, 2015; Kahle et al., 2008), which can lead to human exposure via food and drinking water or in an occupational setting (Khay et al., 2008; Li et al., 2012). Ketoconazole and clotrimazole are used as human pharmaceuticals and are classified as pregnancy category C (i.e. risk cannot be ruled out) by the FDA due to the observed embryotoxic effects in animal studies (Cummings et al., 1997; Draskau et al., 2021; Patel et al., 2021).

To assess the effect on reproduction, worms were exposed to the azole chemicals from the fourth larval stage (L4), a key period for spermatogenesis and after which the worm begins reproduction. The total cumulative number of viable offspring in the negative control was 269, with a similar brood size observed in worms exposed to the vehicle control (0.2% methanol; Figure 1B ). Exposure to clotrimazole and ketoconazole did not affect total brood size at any of the concentrations tested. In contrast, brood size was reduced upon exposure to 100µM triflumizole, with worms producing an average of 153 viable progeny, a reduction of 43% compared to both negative and vehicle controls ( Figure 1B ).

To explore the impact on post-embryonic development, worms were exposed to the 3 azoles from hatching (L1 stage) to L4 stage. All control worms reached the L4 stage after 48 hours at 20 ^o^ C. Triflumizole exposed worms had normal development at 10µM, but there was a delay observed in 21% of worms at 30µM triflumizole ( Figure 1C ). Of those worms grown in the presence of 100µM triflumizole, 59% died before reaching the L4 stage and of those that survived only 5% reached the L4 stage, with the remainder of nematodes being significantly under-developed. Clotrimazole had a minor impact on development at 30µM, where just 3% of the worms failed to reach L4 stage after 48 hours. Exposure to 100µM clotrimazole caused 72% of the worms to be younger than L4 after 48 hours, but no worms died at this concentration. Exposure to ketoconazole had no effect on developmental rate at all concentrations tested.

Together, these data show that triflumizole and clotrimazole have significant effects on development, with triflumizole impacting both reproduction and development in *C. elegans* . This is in line with triflumizole effects found in rodents (Li et al., 2012; Martin et al., 2011) and zebrafish embryos (Bai et al., 2022). However, further exploration of the mechanism of action is needed to support the predictive value of the DART observed in *C. elegans* for mammalian DART. This is especially important for the identification of chemicals that cause DART through an endocrine-mediated effect, i.e., endocrine disrupting chemicals (EDCs). While *C. elegans* does not have an endocrine system, it does respond to EDCs and contains estrogen binding proteins (Hood et al., 2000). Nematode homologs of the mammalian estrogen receptor and androgen receptor have been identified in *C. elegans* (Jeong et al., 2019), which further supports the potential of the nematode to screen chemicals for their endocrine disrupting potential. More recently, *C. elegans* has been used to study the effects of EDCs on various endpoints including fertility and growth (Chen et al., 2019; Jeong et al., 2019; Wittkowski et al., 2019), indicating that the worm may serve as a valuable tool to offer insights into human relevant adverse health effects.

Our knowledge concerning the impact to humans of exposure to azoles during sensitive life stages, such as *in utero* exposure during pregnancy, remains limited. For example, clotrimazole is considered safe to use to treat oral and vaginal candidiasis during pregnancy (Mogensen et al., 2017; Munkboel et al., 2019), but as patients can self-medicate, exposure levels are ambiguous. While exposure to clotrimazole resulted in abnormal plasma steroid hormone concentrations in maternal and foetal rats, no morphological differences were observed in the reproductive system and it was unclear if these translated to fertility defects (Draskau et al., 2021). Our *C. elegans* data demonstrates that while fertility is not directly impacted, exposure to clotrimazole can have detrimental effects on the development of progeny at elevated concentrations. Further exploration of internal exposure levels of the azoles *in C. elegans* are needed to translate the effect concentrations to the human and/or rodent situation. Still, our data supports the fact that azole fungicides should be used with caution during pregnancy.

Together, our results support the use of the relatively fast and low-cost *C. elegans* DART assay as a tool to screen for reproductive and developmental effects of chemical exposure.

## Methods

Strain maintenance and synchronisation: All experiments were conducted with the wild type *C. elegans* strain N2 var. Bristol. Nematodes were maintained on Nematode Growth Media (NGM) agar according to standard protocols (Brenner, 1974), with OP50 *E. coli* as the bacterial food source. To synchronise the nematodes, standard bleaching was performed (Stiernagle, 2006). Gravid adults were collected and treated with alkaline hypochlorite solution. After washing, the eggs were left to hatch overnight at room temperature (19-21 ^o^ C) in the absence of food in M9 buffer. The following day, synchronised L1s were plated out onto NGM plates with OP50 *E. coli* and left to develop to L4s at 20 ^o^ C. All experiments take place at 20 ^o^ C.

Brood size assay: Nematodes were age synchronised and grown to the L4 stage on OP50 seeded NGM. NGM was supplemented with the azoles to a final concentration of 10, 30 or 100 µM, or the methanol vehicle control. Spiked NGM (2 ml) was distributed into 12-well plates. *E. coli OP50* (40 µl) was added to the centre of each well and allowed to dry inside a laminar flow hood at room temperature (21 ^o^ C). Nematodes (wild type N2 strain) were synchronised to L1 larvae using standard methods and when at the L4 stage, a single worm was transferred into each well and the plates incubated at 20 ^o^ C. The parental worm was transferred daily until the reproductive period was complete (5 days) and the number of viable offspring counted. Daily reproductive output and total brood size was determined for each condition. Counting of the offspring was performed manually across 2 independent biological replicates, each with at least 10 individuals, and combined.

Developmental assay: Age synchronised nematodes at the L1 stage were added to OP50 seeded NGM plates supplemented with azoles (10, 30 or 100 µM) or vehicle control (methanol). Plates were left at 20 ^o^ C for 48 hours, after which the worms were assessed for developmental stage (Hughes et al., 2022). The L4 stage is easily recognisable by the formation of the vulva, and worms were then classified as older (i.e., containing eggs) or younger. For each condition at least 50 worms were assessed and the data analysed to find the percentage of worms at each developmental stage.

Statistical analysis: Statistical analysis was performed on GraphPad Prism 9 and Microsoft Excel. For the brood size, a 2-tailed 2-sample t-test was performed, comparing exposed conditions to the controls. A Chi-square test was performed for the developmental delay.

## Reagents

Wild type *Caenorhabditis elegans* strain N2 (var. Bristol) and *E. coli* OP50 were provided by the GCG, funded by the NIH Office of Research Infrastructure Programs (P50 OD010440). Azoles were all supplied from Sigma (Triflumizole #332611; Clotrimazole #C6019; Ketoconazole #K1003) and made to a 50 mM stock solution in methanol. In all cases, the final solvent control concentrations in experiments were 0.2% methanol.
